# Changes in Lithium Levels in Bees and Their Products Following Anti-*Varroa* Treatment

**DOI:** 10.3390/insects12070579

**Published:** 2021-06-25

**Authors:** Éva Kolics, Zsófi Sajtos, Kinga Mátyás, Kinga Szepesi, Izabella Solti, Gyöngyi Németh, János Taller, Edina Baranyai, András Specziár, Balázs Kolics

**Affiliations:** 1 Festetics Bioinnovation Group, Institute of Genetics and Biotechnology, Georgikon Campus, Hungarian University of Agriculture and Life Sciences, H-8360 Keszthely, Hungary; kolicseva@gmail.com (É.K.); petrovicsnemkk@gmail.com (K.M.); szepesikinga66@gmail.com (K.S.); izabella.solti@gmail.com (I.S.); drnemethgyongyi@gmail.com (G.N.); taller.janos@uni-mate.hu (J.T.); 2 Kolics Apiaries, H-8710 Balatonszentgyörgy, Hungary; 3 Doctoral School of Chemistry, University of Debrecen, H-4032 Debrecen, Hungary; sajtos.zsofi@science.unideb.hu; 4 Atomic Spectrometry Partner Laboratory, Department of Inorganic and Analytical Chemistry, Faculty of Science and Technology, University of Debrecen, H-4032 Debrecen, Hungary; baranyai.edina@science.unideb.hu; 5 Balaton Limnological Research Institute, ELKH, H-8237 Tihany, Hungary; specziar.andras@blki.hu

**Keywords:** lithium chloride, beeswax, honey, chemical residues, *Apis mellifera*, *Varroa destructor*

## Abstract

**Simple Summary:**

Varroosis caused by the ectoparasitic mite *Varroa destructor* has been the biggest threat to managed bee colonies over recent decades. Chemicals available to treat the disease imply problems of resistance, inconsistent efficacy, and residues in bee products. Recently, alongside novel compounds to defeat the pest, lithium chloride has been found to be effective. In this study, we found that lithium treatments leave beeswax residue-free. The possibility of decontamination in adult bees, bee bread, and uncapped honey was revealed. On the other hand, ripe honey was found to be affected by lithium administered via feeding. Case studies are necessary to uncover the level of exposition in harvested honey to estimate its potential risk once it becomes a registered veterinary medicine.

**Abstract:**

The biggest threat to beekeeping is varroosis caused by the mite *Varroa destructor*. Chemicals available to treat this fatal disease may present problems of resistance or inconsistent efficacy. Recently, lithium chloride has appeared as a potential alternative. To date, the amount of residue lithium treatments may leave in honeybee products is poorly understood. Honeybees were fed with 25 mM lithiated sugar syrup, which was used in earlier studies. The accumulation and elimination of the lithium were monitored in bees and their products for 22 days. Lithium concentration increased in the entire body of the bees to day 4 post-treatment and then recovered rapidly to the control level. Lithium exposure was found to affect uncapped honey in the short term (<16 days), but ripe (capped) honey measured at the end of the trial remained affected. On the other hand, lithium treatment left beeswax lithium-free. Based on these data, we propose that comprehensive research on harvested honey is needed to decide on the veterinary use of lithium.

## 1. Introduction

Hive products are associated with nutritional benefits and are value-added products of the human food chain. However, honey, beeswax, and bee bread may be exposed to pesticides as a consequence of anti-*Varroa* treatment.

The overwhelming majority of pollination carried out by honeybees (*Apis mellifera*) is performed by managed bee colonies [1]. The biggest threat to the honeybee worldwide is varroosis, which involves virus diseases transmitted by the ectoparasite *V. destructor* originally parasitizing a closely related species (*A. cerana*). If left untreated, mites can kill an entire colony within one or two years [2,3], but, in areas of a high density of honeybee colonies, it might occur within an apicultural season. Controls currently in practice based on synthetics can be adequate but are restricted to a few chemicals such as amitraz, coumaphos, flumethrin, and fluvalinate, the formulations of which, however, are demonstrated to lead to the risk of development of resistance [4,5]. As a result, they offer a limited possibility of mite eradication in the foreseeable future. With the exception of oxalic acid, one of the most widely used varroacides [6,7], alternatively used essential oils or organic acids in some instances may be inconsistent in efficacy [8]. Alongside novel approaches (RNAi) to treating varroosis, lithium salts were found to be effective in eradicating *V. destructor* in vitro [9]. Although publications concerning the effects of lithium on harnessed bee individuals are available, these remain restricted to physiological studies [10,11,12].

Concerning other invertebrates (e.g., sea urchins, marine polychaete worms), disturbances in embryonal development were raised in relation to lithium compounds [13]. Interestingly, however, beneficial effects of lithium on longevity were detected in adult individuals of *Drosophila* [14]. For both honeybee adults and brood, adverse effects on the longevity of lithiated sugar syrup administration is reported but restricted mainly to in vitro trials [9,15]. Furthermore, it is of note that a freely moving bee might react differently to aversive compounds. Being an obligatory social organism, a honeybee colony could actively reject aversive substances [12]. Moreover, feeding sugar syrup infused with varroacide is not typically the way of administering an anti-*Varroa* treatment in apicultural practice [16].

Lithium chloride (PubChem CID: 433294) may provide an effective, commercially available, and relatively cheap alternative, and therefore, it may be increasingly applied as an unregulated veterinary medicine [16,17,18,19]. Despite its potential to treat varroosis in the short term, only a few studies are dealing with the consequences of lithium treatments on honey and other bee products [15,20]. However, with honey and beeswax being the most remarkable hive products worldwide, human exposure to lithium when it is used needs to be extensively studied in the apiculture-related food chain to evaluate its impacts before it ever becomes a registered veterinary medicine.

We aimed to understand the consequences of anti-*Varroa* treatment using lithium chloride feeding, monitoring the changes of the lithium level in the bees and their most important products.

## 2. Materials and Methods

### 2.1. Colony Setup and Samplings of Biological Materials and Apicultural Products

The experiment was started in early October 2018 in Hungary (Keszthely, 46°45′55.6″ N, 17°14′52.6″ E), excluding outer nectar flow. Carniolan (*A. m. carnica*) colony splits were populated into four hives (local type). On the same day (day 6), colonies were transferred away from their flight range into a dark room and kept for five days. Apart from one frame originating from the donor colonies, each hive was equipped with wax foundations (day 3) only. After making sure the colonies were queenright, they were placed outdoors in the evening of day 1. Hives were set at least 3 m away from each other, with geographical landmarks to prevent drifting.

Sampling was initiated on day zero. Before treatment, bees and their products were sampled to measure control lithium concentrations in the four hives. Then, the frame originating from the donor colonies containing the honey and bee bread store from pre-treatment was discarded from each hive. The colonies were subsequently fed with one liter of 1:1 sucrose syrup containing 25 mM lithium chloride (126.5 mg kg^−1^ Li+) [9].

Sampling was carried out in a standardized manner to prevent cross-contamination of the hive products as follows. First, bottom boards were cleaned to collect hive debris. Adult bees (25 workers, mixed of age, from each hive in each occasion) were collected from the bee space of the hives to make a pooled sample. To be able to sample beeswax secreted under lithium exposure from the hive, the colony was forced to build brace combs (about 10 × 10 cm in size). Cells from which the bee bread was collected (2 g from each hive) were marked to prevent their re-sampling (except the pre-treatment control originating from the donor colony). Taken from all combs, 30 mL honey was collected from each hive on each sampling occasion. Samplings were carried out on day 0 (pre-treatment control), and days 1, 4, 8, 16, and 22 (post-treatment) for hive debris (*n* = 24), bees (*n* = 24) divided later into three body parts (head, thorax and abdomen, and legs), brace combs (*n* = 19), bee bread (*n* = 24), and uncapped (unripe) honey (*n* = 24).

**Figure 1:**
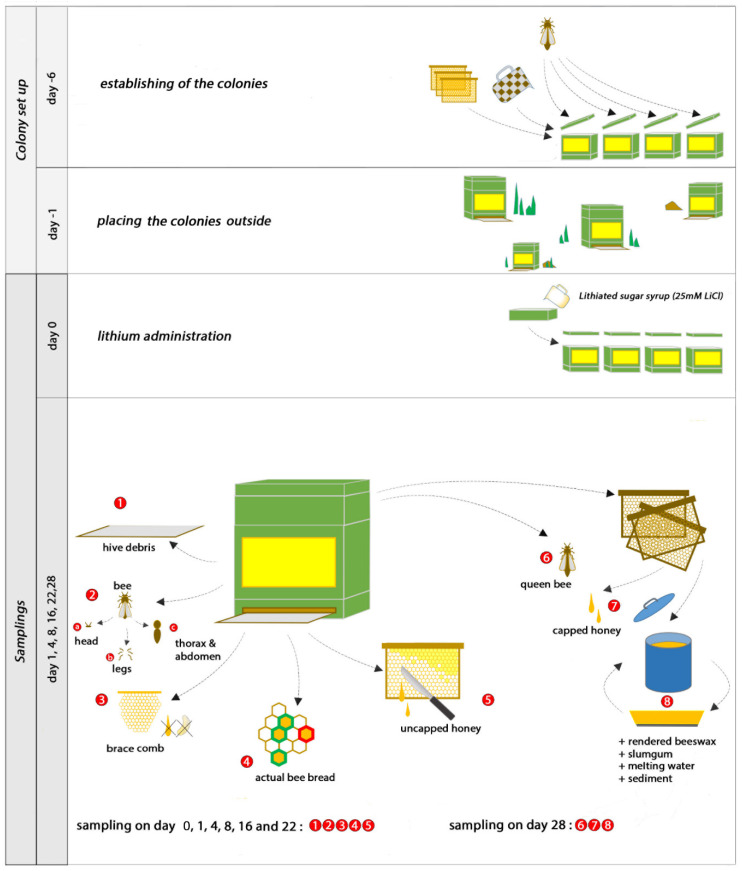
Experimental design and samplings.

The experiment was terminated on day 28. Queens were killed to examine their whole body (*n* = 4). Mature, capped honey was sampled (*n* = 4). Beeswax was rendered from the combs; during this process, slumgum was collected (*n* = 4). Furthermore, sediments (*n* = 4) of the wax cakes and the melting waters (*n* = 4) in which the wax (*n* = 4) was processed were collected. Altogether, 139 different samples were collected. An overview of the whole sampling process is visualized in Figure 1.

### 2.2. Sample Preparation

Samples were stored at −5 °C in plastic tubes before the sample pre-treatment process. Bees’ heads were measured separately since pharyngeal glands produce and excrete royal jelly, presenting the food for honeybee larvae. The lithium content of the legs was measured separately.

Bees were separated into the three main parts according to the details above: approx. 75 mg for the head, and the same amount for the legs, and approx. 500 mg for the thorax & abdomen in each sampling. The body parts, as well as the exact known weight of honey and beeswax (0.5 g of each), bee bread, and hive debris (0.1 g of each), were measured on an analytical balance (ES 225SM-DR, Precisa, Dietikon, ZH, USA) into 50 mL glass beakers. Samples were dried at 50 °C to constant weight in an electric drying cabinet.

Dried samples were wet digested in the same vessels by the mixture of 4.0 mL 65% (m/m) HNO_3_ (reagent grade, Scharlau, Germany) and 1.0 mL 30% (m/m) H_2_O_2_ (reagent grade, Merck, Kenilworth, NJ, USA) to evade the cross-contamination from changing glassware. Digested samples were transferred without loss into volume calibrated plastic centrifuge tubes and diluted up to the volume of 10.00 mL with ultrapure water (Synergy UV, Sigma-Aldrich, St. Louis, MO, USA). Solutions were kept at room temperature before further elemental analysis. Each piece of glassware used was decontaminated by immersion in a 1:5 HNO**_3_**: H**_2_**O solution for 24 h and rinsed with deionized water before use.

### 2.3. Analytical Measurements

The quantitative analysis of the lithium content in the different samples was carried out by microwave plasma atomic emission spectrometry (MP-AES 4200, Agilent Technologies, Santa Clara, CA, USA). The plasma gas was continuously supplied during measurement by a nitrogen generator (4107, Agilent Technologies). The MP-AES instrument operates with a vertical torch alignment together with an axial observation position. As well as sample solutions, standards were introduced by autosampler (SPS, Agilent Technologies) with 30 s of rinsing between each with 0.1M HNO**_3_** prepared in ultrapure water. The MP-AES operating conditions and measurement parameters are indicated in Table 1. Lithium standard stock solution of 1000 mg L^−1^ (Scharlau, Germany) was used to prepare the 5-point calibration series. The limit of detection (LOD) was defined as 0.3246 µg kg^−1^ at the applied wavelength of 610.365 nm (the measurement parameters are summarized in Table 1).

The following formula calculated the LOD: LOD = (3 × s)/S where s is the standard deviation of 15 blank samples, and S is the specificity (slope of the calibration curve). The results of the elemental analysis were given on a dry mass basis.

### 2.4. Statistical Analysis

To analyze the effect of LiCl treatment on the Li concentration of bees (head, thorax and abdomen, and leg were analyzed separately), honey, bee bread, beeswax and hive debris (response variables), we used linear mixed models (LMMs) in Statistica 8.0 (http://www.statsoft.hu) (accessed on 19 May 2021). Prior to analysis, Li concentration data were log10 transformed to improve normality. The LMMs included sampling time as a fixed factor representing pre-treatment (control, at day 0) and post-treatment measurements (days 1, 4, 8, 16, and 22). In order to account for repeated measures, the hive was included as a random factor. Differences among means were identified using Tukey HSD post hoc tests when the model fixed effect was significant.

## 3. Results and Discussion

### 3.1. Lithium Level Returns to Normal Values in Adult Bees

**Figure 2:**
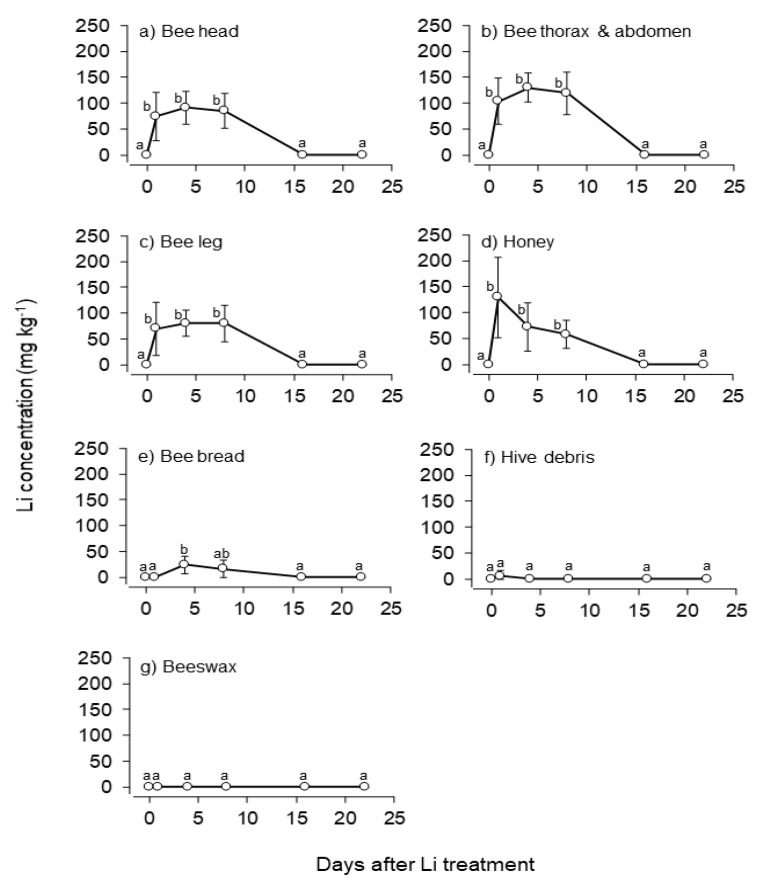
Post-treatment changes in lithium concentration in hive products and bees. Linear mixed model analysis (LMM) revealed a significant increase in Li concentrations (mean ± SD) in bee head (**a**), thorax and abdomen (**b**), and leg (**c**), as well as in honey (**d**) between 1 and 8 days after LiCl treatment, followed by a return of concentrations to the control level after 16 days post-treatment. Only a small increase in Li concentration was revealed in bee bread (**e**), and only on the 4th day post-treatment, and no increase in Li concentration at all was detected in hive debris (**f**) or beeswax (**g**). For LMM statistics, see Table 2. Plotted values not sharing any index letter are statistically different at *p* < 0.05 (Tukey HSD post hoc test). Note that the threshold of detecting Li was 0.0003 mg kg^−1^.

Feeding lithium syrup at a concentration (25 mM) applied in earlier studies [9,15,20] resulted in an average lithium peak of 130.13 mg kg^−1^ (average of the hives) in bees’ bodies (thorax and abdomen), with an absolute maximum value of 167.71 mg kg^−1^ in hive 1, on day 4. Lithium concentration decreased in all body parts of the bees from day 4 post-treatment (Table 2, Figure 2). This pattern may be consistent with the findings of Prešern and colleagues**,** who revealed that in bee larvae, lithium level started to drop on day 3 post-treatment [15]. By day 22 post-treatment, lithium level showed full recovery to the pre-treatment control level (0.15 mg kg^−1^ on average; Figure 2). Data from the present study indicate that adult bees seem to be able to excrete lithium at the colony level.

Residues were found in the entire body of bees, reaching the legs as well, the parts of the body in which lithium may have been transferred via the hemolymph. Therefore, all parts of the bees’ bodies may be eligible to estimate changes in the lithium level of the colony. We hypothesize that the Li concentration peak measured in the bees and their offspring might help to predict the timeframe of treatment efficacy for future research in this field.

A sampling of the queens was only possible by killing the individuals. Therefore, the queens were measured only at the termination of the trial (day 28), revealing no detectable lithium. No signs of an attempt to refuse the queens by the worker bees, known as supersedure, were observed in the whole period of the experiment. We conclude that lithium has no detrimental effect on the queen, at least in the short term.

Hive debris usually contains wax particles and may contain bee parts, traces of honey, or pollen in a variable composition. Nonetheless, it revealed no response to LiCl treatment (Table 2, Figure 2). Despite being readily available without opening the hive, the debris does not appear to be suitable for collecting information about the lithium level in the hive.

### 3.2. Bee Bread Is the Least Affected of Beekeeping Products

Of the bee products in which lithium appeared, the stored, fermented pollen (known as bee bread) was found to be the least exposed to lithium contamination (Table 2).

Bee bread is commercialized for its beneficial nutritional and therapeutic properties. However, collecting it for human food is time-consuming and suffers from limitations [21]. Similar to other samples investigated, lithium peaked on day 4 with a lithium level of 28.11 mg kg^−1^ (Figure 2). Representing four hives but a single time measurement, a similar value (30.75 mg kg^−1^) was reported for day 4 post-treatment by Prešern and collegues [15]. Bee bread is the primary protein resource that bees utilize, especially for feeding larvae and adults. High lithium exposure may adversely affect the development of the larvae **[15]**. Furthermore, increased mortality in the lithium-treated colonies was recorded. Thus, lithium treatment may have an impact on colony reproduction. Nevertheless, reduced lithium levels were measured in 5-day-old larvae three days after the lithium culmination (on day 7 post-treatment). [22].

Our data support the rapid decrease of LiCl in the bee bread after the peak caused by treatment (Figure 2). Lithium concentration recovery in adult bees and in the diet of larvae enables the brood to be less exposed over time. We propose that the possible adverse effects of lithium might be compensated for or minimized by applying it only in naturally or artificially induced brood-free or brood-poor periods. It should be noted, however, that the veterinary use of LiCl has not been authorized yet. Further research is needed to accurately determine a low-risk timing or a withholding period in queen rearing.

### 3.3. Lithium Treatment Leaves Beeswax Unaffected

In brace combs, representing the wax secreted directly by the bees, no lithium was detected in any samples (*n* = 19) at any sampling time (Figure 2). No lithium was detected in any lumps of wax (*n* = 4) rendered from the old combs of the hives, either. Moreover, in by-products of the wax rendering process from old combs such as the slumgum, sediment, and the melting water, no lithium content has been confirmed. These facts are of great importance as comb wax is commonly recycled in apicultural practice; recycled comb is used to make beeswax foundations and widely distributed to beekeepers.

Our finding is significant also because other commonly used acaricides such as amitraz [23], coumaphos [24], tau fluvalinate [25], flumethrin [26], and thymol [27] affect the beeswax.

### 3.4. Lithium Levels Decrease during Dehydration, but Residues May Remain in the Ripe Honey

Honey, the most important apicultural product, represents aggregate honey taken from thousands of honey storage sites from capped and uncapped cells.

Uncapped cells initially contain freshly collected unripe nectar, which undergoes the process of dehydration and transposition to cells to be filled into the vicinity of the brood, where they are then capped by the bees. In the present study, uncapped honey served to uncover the kinetics of lithium within the hive, whilst capped honey was separately handled to represent the store to be harvested at the termination of the experiment.

Treatment with lithiated sugar syrup containing 126.5 mg kg^−1^ LiCl (25 mM) affected the uncapped honey considerably in the short term (Table 2, Figure 2). The highest lithium content in the honey was measured on day 1 (Figure 2). Despite the dehydration process of the honey carried out by the bees, the concentration of lithium started to decrease from day 4 post-treatment. Lithium concentration in uncapped honey showed full recovery to the control level (below 0.25 mg kg^−1^) by day 22. Based on the obtained data, the possibility of decontamination of uncapped honey has been confirmed. This hive product, being the transposed honey stock of the colony, is most affected by lithium. Incoming nectar or sugar syrup is processed and exposed to transposition from being passed from bee to bee several times. It is hypothesized that one possible point of lithium depletion may occur via the bees.

In the capped, ripe honey, a value corresponding to one-fifth of the initial lithium syrup concentration (22.40 mg kg^−1^) was measured on average at the termination of the experiment (day 28). This amount may be comparable to the natural lithium trace element content measured in honey so far [28]. Honey exerts positive nutritional and health effects if consumed at high doses of 50 to 80 g per daily intake [29]. Considering it as a proposed intake, capped honey from the present trial would equal 1.12–1.79 mg lithium. This amount can be achieved from other alimentary products as a daily lithium intake [30,31,32]. It is of note that hardly any similar modes of administration are used in which the anti-*Varroa* active ingredient is applied via a large amount of sugar syrup (e.g., feeding), since it will inevitably induce an elevated residue level in the honey store. Although a single treatment is not likely to result in an alarming level in ripe honey, a trickling mode of administration may be preferred once lithium is registered as a veterinary medicine.

## 4. Conclusions

In this study, the progress of contamination and the possibility of subsequent elimination of lithium in the most important bee products and adult bees were investigated in situ after lithiated sugar syrup feeding. Unlike commonly used varroaicides, lithium treatment left beeswax unexposed as a clear positive property of lithium. On the other hand, it was revealed that lithium could contaminate ripe honey. Despite inducing the pollution of the honey by feeding the bees lithiated syrup, lithium levels remained under the level of commercialized honey, which naturally possess higher lithium content (38–110 mg/kg^−1^ [28,33]. Currently, no maximum residue levels (MRL) exist for lithium, nor it is recognized as veterinary medicine. More extensive research is needed to determine lithium residues under field conditions in harvested honey and bee bread, as well as to determine a waiting time after the Li treatment. Further experiments are necessary to reveal how application methods like trickling [16] would affect the appearance of residues in honey, especially if performed repeatedly against the devastating pest *V. destructor*.

## Figures and Tables

**Table 1:** Analytical measurement parameters.

Replicates Pump Speed	3 15 rpm
Uptake time	15 s
Rinse time	30 s
Stabilization time	15 s
Read time	3 s
Nebulizer pressure	240 kPa
Wavelength	610.365 nm

**Table 2:** Effect of lithium in bees and bee products in factor of time. Analysis of log-transformed Li concentration data using linear mixed models revealed a significant effect of time relative to the LiCl treatment (control: pre-treatment (day 0); treated: 1, 4, 8, 16, and 22 days post-treatment) of honeybee colonies on lithium concentration of the bees, the honey, and the bee bread, but not that of the wax and the hive debris. Results of Tukey HSD post hoc tests are shown in Figure 2.

	Main Effects					Overall Model			
	Factor	Effect type	d.f. (Effect, Error)	F	*p*	R^2^_adj._	d.f. (Model, Residual)	F	*p*
Bee head	day	fixed	5, 15	154.4	<0.001	0.971	8, 15	96.8	<0.001
	hive	random	3, 15	0.8	0.529				
Bee thorax and abdomen	day	fixed	5, 15	395.1	<0.001	0.988	8, 15	247.9	<0.001
	hive	random	3, 15	2.5	0.102				
Bee leg	day	fixed	5, 15	156.2	<0.001	0.971	8, 15	97.9	<0.001
	hive	random	3, 15	0.7	0.592				
Honey	day	fixed	5, 15	57.3	<0.001	0.925	8, 15	36.5	<0.001
	hive	random	3, 15	1.7	0.210				
Bee bread	day	fixed	5, 15	6.6	0.002	0.543	8, 15	4.4	0.006
	hive	random	3, 15	0.8	0.512				
Hive debris	day	fixed	5, 15	1.0	0.451	0.000	8, 15	1.0	0.474
	hive	random	3, 15	1.0	0.418				

## Data Availability

Not applicable.
